# Draft Genome Sequence of Saccharomyces cerevisiae Strain Awamori Number 101, Commonly Used to Make Awamori, a Traditional Spirit, in Okinawa, Japan

**DOI:** 10.1128/MRA.01414-20

**Published:** 2021-06-24

**Authors:** Masatoshi Tsukahara, Kotaro Ise, Maiko Nezuo, Haruna Azuma, Takeshi Akao, Hirohide Toyama

**Affiliations:** a Biojet Company Ltd., Uruma, Okinawa, Japan; b National Research Institute of Brewing, Higashihiroshima, Hiroshima, Japan; c Department of Bioscience and Biotechnology, Faculty of Agriculture, University of the Ryukyus, Sembaru, Okinawa, Japan; University of California, Riverside

## Abstract

We report here the draft genome sequence for Saccharomyces cerevisiae strain Awamori number 101, an industrial strain used for producing awamori, a distilled alcohol beverage. It was constructed by assembling the short reads obtained by next-generation sequencing. The 315 contigs constitute an 11.5-Mbp genome sequence encoding 6,185 predicted proteins.

## ANNOUNCEMENT

Awamori is a traditional spirit of the Okinawa (Ryukyu) region in Japan. It is made from steamed rice with cooperative action of a filamentous fungus, Aspergillus luchuensis (1), and a yeast, Saccharomyces cerevisiae. In the awamori-making process, the enzymes secreted from the mycelia of *A. luchuensis* that is cultivated on steamed rice, called awamori koji, degrades rice starch into glucose. S. cerevisiae continuously converts the decomposing rice materials into ethanol and a small amount of the other flavor compounds, followed by distillation. In some cases, the distillate is aged to become an awamori product. S. cerevisiae strain Awamori number 101 (AW101) (2) was developed by spontaneous mutation as an improved variant of Awamori number 1 (AW1), which was isolated from awamori mash (3). AW101 is considered to have the distinct characteristics of awamori production, such as high alcohol productivity and favorable sensory properties, and is used in commercial brewing. Accordingly, today, it is commonly used in nearly all awamori distilleries. Here, we report the whole-genome sequence of S. cerevisiae strain AW101.

S. cerevisiae strain AW101 maintained in the Okinawa Regional Taxation Office was used. Cells were cultured in a nutrient-rich YPD medium (2% glucose, 1% yeast extract, 2% peptone). Genomic DNA was extracted, and paired-end libraries were constructed using a Nextera DNA library preparation kit (Illumina, San Diego, CA, USA). Genome sequencing was performed on the Illumina MiSeq sequencing platform. The data obtained using the MiSeq reagent kit 2 (250-bp paired-end reads) and the MiSeq reagent kit 3 (300-bp paired-end reads) have been merged. Quality assessment and adapter trimming were performed using CLC Genomics Workbench version 8.5.1 (Qiagen). The adapter sequences were deleted; trimming was done based on a quality score (limit, 0.05). Ambiguous nucleotides (maximum of 2) were also trimmed, and the resultant sequences comprised a total of 8,623,236 reads. *De novo* assembly was performed using the “guidance only reads” function of CLC Genomics Workbench version 8.5.1 (Qiagen). The S. cerevisiae S288c reference genome (NCBI RefSeq number GCF_000146045.2) was used as guidance; it was not used to create the de Bruijn graph and subsequent contig sequences but was only used to resolve ambiguities in the graph. The overall coverage was estimated to be 174.6×. The final assembly consisted of 351 contigs and an *N*_50_ contig size of 75,854 bp, and the maximum contig length was 297,490 bp. The genome size of S. cerevisiae strain AW101 was found to be 11,455,750 bp with a G+C content of 38.2%. The gene predictions were conducted by using Augustus version 2.5.5 (4) with the available training set for S. cerevisiae, which showed that the draft genome contains 6,185 predicted open reading frames (ORFs).

### Data availability.

The reads and the assembled genome sequences of S. cerevisiae strain AW101 have been deposited in DDBJ/EMBL/GenBank under BioProject number PRJDB4903 with accession number BNHH00000000. This first version of the project consists of sequences BNHH01000001 to BNHH01000351.

## References

[1] Hong SB, Lee M, Kim DH, Varga J, Frisvad JC, Perrone G, Gomi K, Yamada O, Machida M, Houbraken J, Samson RA. 2013. *Aspergillus luchuensis*, an industrially important black *Aspergillus* in East Asia. PLoS One 8:e63769. doi:10.1371/journal.pone.0063769. 23723998 PMC3665839

[2] Shinzato S, Miyagi T, Takaesu C, Maruyama S. 1989. Characteristics of a non-foaming awamori yeast isolated from Awamori no.1 yeast. J Brew Soc Jpn 84:121–123. doi:10.6013/jbrewsocjapan1988.84.121.

[3] Tamaki T, Ninjoji A, Imamura T, Harada T. 1981. Isolation and selection of the excellent awamori yeast. J Soc Brew Jpn 76:59–62. doi:10.6013/jbrewsocjapan1915.76.59.

[4] Stanke M, Diekhans M, Baertsch R, Haussler D. 2008. Using native and syntenically mapped cDNA alignments to improve *de novo* gene finding. Bioinformatics 24:637–644. doi:10.1093/bioinformatics/btn013. 18218656

